# Establishment of an *in vitro* transcription system for *Peste des petits* ruminant virus

**DOI:** 10.1186/1743-422X-9-302

**Published:** 2012-12-05

**Authors:** Mohammad Yunus, Melkote S Shaila

**Affiliations:** 1 Department of Microbiology and Cell Biology, Indian Institute of Science, Bangalore, 560012, India

**Keywords:** *Peste-des-petits ruminants* virus (PPRV), Transcription reconstitution, RNA dependent RNA polymerase, Morbillivirus

## Abstract

**Background:**

*Peste-des-petits ruminants* virus (PPRV) is a non segmented negative strand RNA virus of the genus Morbillivirus within *Paramyxoviridae* family. Negative strand RNA viruses are known to carry nucleocapsid (N) protein, phospho (P) protein and RNA polymerase (L protein) packaged within the virion which possess all activities required for transcription, post-transcriptional modification of mRNA and replication. In order to understand the mechanism of transcription and replication of the virus, an *in vitro* transcription reconstitution system is required. In the present work, an *in vitro* transcription system has been developed with ribonucleoprotein (RNP) complex purified from virus infected cells as well as partially purified recombinant polymerase (L-P) complex from insect cells along with N-RNA (genomic RNA encapsidated by N protein) template isolated from virus infected cells.

**Results:**

RNP complex isolated from virus infected cells and recombinant L-P complex purified from insect cells was used to reconstitute transcription on N-RNA template. The requirement for this transcription reconstitution has been defined. Transcription of viral genes in the *in vitro* system was confirmed by PCR amplification of cDNAs corresponding to individual transcripts using gene specific primers. In order to measure the relative expression level of viral transcripts, real time PCR analysis was carried out. qPCR analysis of the transcription products made *in vitro* showed a gradient of polarity of transcription from 3’ end to 5’ end of the genome similar to that exhibited by the virus in infected cells.

**Conclusion:**

This report describes for the first time, the development of an *in vitro* transcription reconstitution system for PPRV with RNP complex purified from infected cells and recombinant L-P complex expressed in insect cells. Both the complexes were able to synthesize all the mRNA species *in vitro*, exhibiting a gradient of polarity in transcription.

## Background

*Peste-des-petits ruminants* virus (PPRV), the causal agent of *peste-des-petits ruminants* (PPR) disease, belongs to the genus *Morbillivirus* within the *Paramyxoviridae* family. The genomes of paramyxoviruses encode six transcription units from which mRNAs coding for both structural and non-structural proteins are generated. Located at the 3’ end of the genome is genomic promoter (GP) region also referred to as the leader region, which is followed by transcription units for N, P, M, F, H and L and ends in the antigenome promoter (AGP) or trailer region at the 5’ end of the genome [1]. The genomic and antigenomic regions are known to play critical roles in initiation of transcription. N protein is essential for encapsidation of the viral genomic RNA which acts as a template for transcription and replication by RNA dependent RNA polymerase (RdRp) complex. L protein itself is not able to recognize and carry out transcription or replication of viral genome encapsidated by N protein. P protein forms an essential component of RdRp and acts as a bridge between N-RNA and L protein [1,2]. For rinderpest virus, our earlier work has shown that the negative sense RNA genome encapsidated by N protein (N-RNA) along with phosphoprotein (P) and large polymerase (L) form the minimal transcription unit of the virus [3-5]. In order to study transcriptional and post-transcriptional activities associated with L protein, a reconstitution system of *in vitro* transcription is required. This has been achieved only for Rinderpest virus among morbilliviruses [5]. Further, employing a minigenome transcription-replication system, it has been shown that mutations in the GDNQ motif in L protein of rinderpest virus results in the inactivation of its polymerase activity *in vitro* as well as *ex vivo*[6].

The present study describes the establishment of an *in vitro* transcription system for PPRV employing purified RNP complex from virus infected cells as well as a reconstituted system employing recombinant L-P complex and N-RNA template. Further, quantitation of transcripts made *in vitro* and in infected cells has been carried out.

## Results

### Components of RNP complex from virus-infected cells

**Figure 1:**
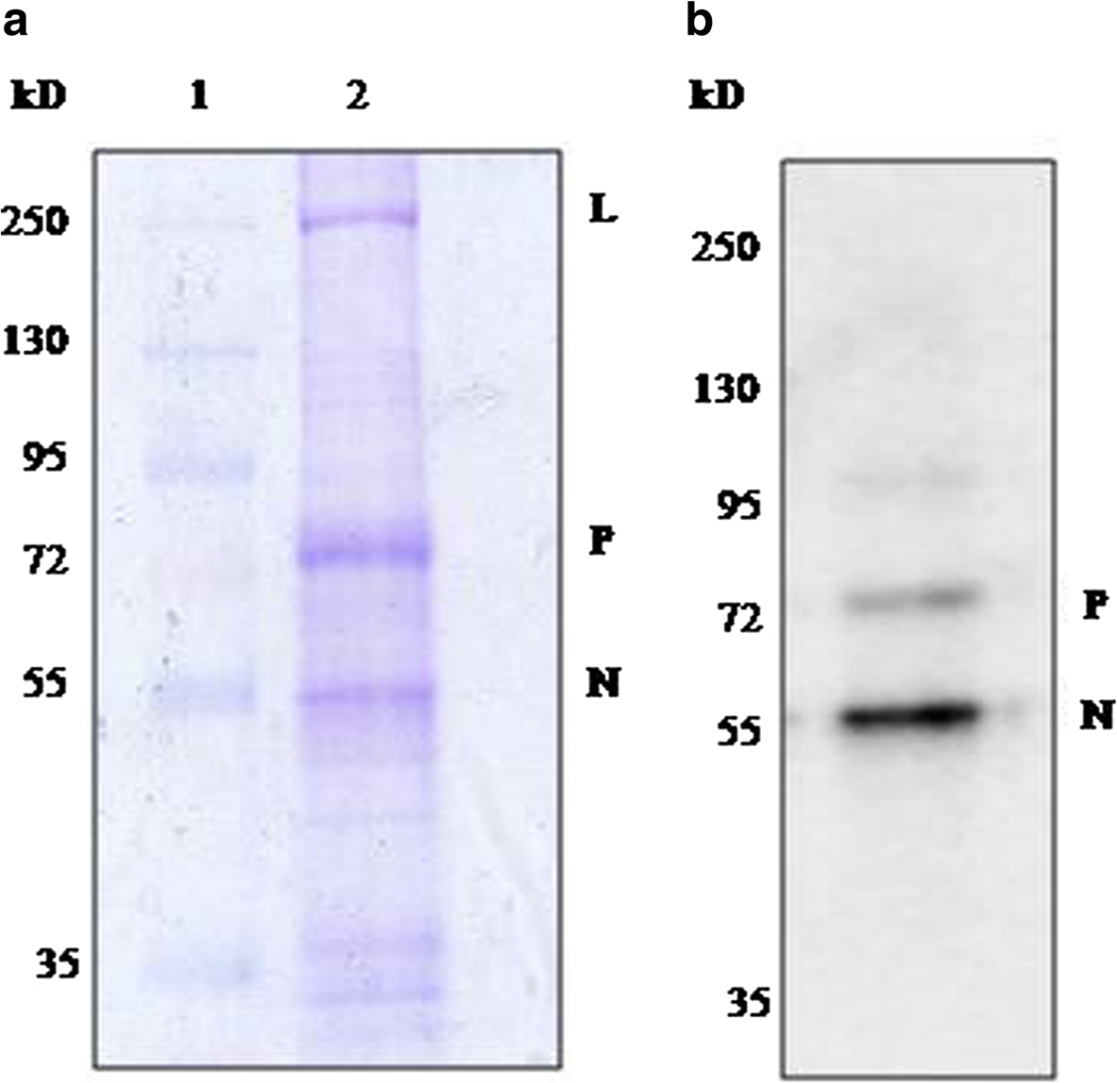
**Protein composition of purified RNP complex from disrupted virus: (a) coomassie staining of RNP complex run on 8% SDS-Polyacrylamide gel.** Lane 1 molecular weight marker (#SM1811, Fermentas), lane 2, 10 μg of RNP complex. (**b**) Western blot of RNP complex with rabbit polyclonal antibody against purified virus (1:5000).

The RNP complex purified from PPRV infected cells was analyzed on SDS-PAGE and visualized by coomassie brilliant blue staining (Figure 1a). The RNP complex consisted of 3 proteins corresponding to the expected molecular weights of N, P and L proteins. The presence of N and P protein in RNP complex was confirmed by western blot analysis using antibody made against purified virus (Figure 1b). L protein could not be detected by western blotting as the L protein amount present in purified virus is very less in comparison to N and P proteins. The result showed that the RNP complex isolated from virus-infected cells contains the RNA polymerase constituents.

### Development of an *in vitro* transcription system with RNP complex

**Figure 2:**
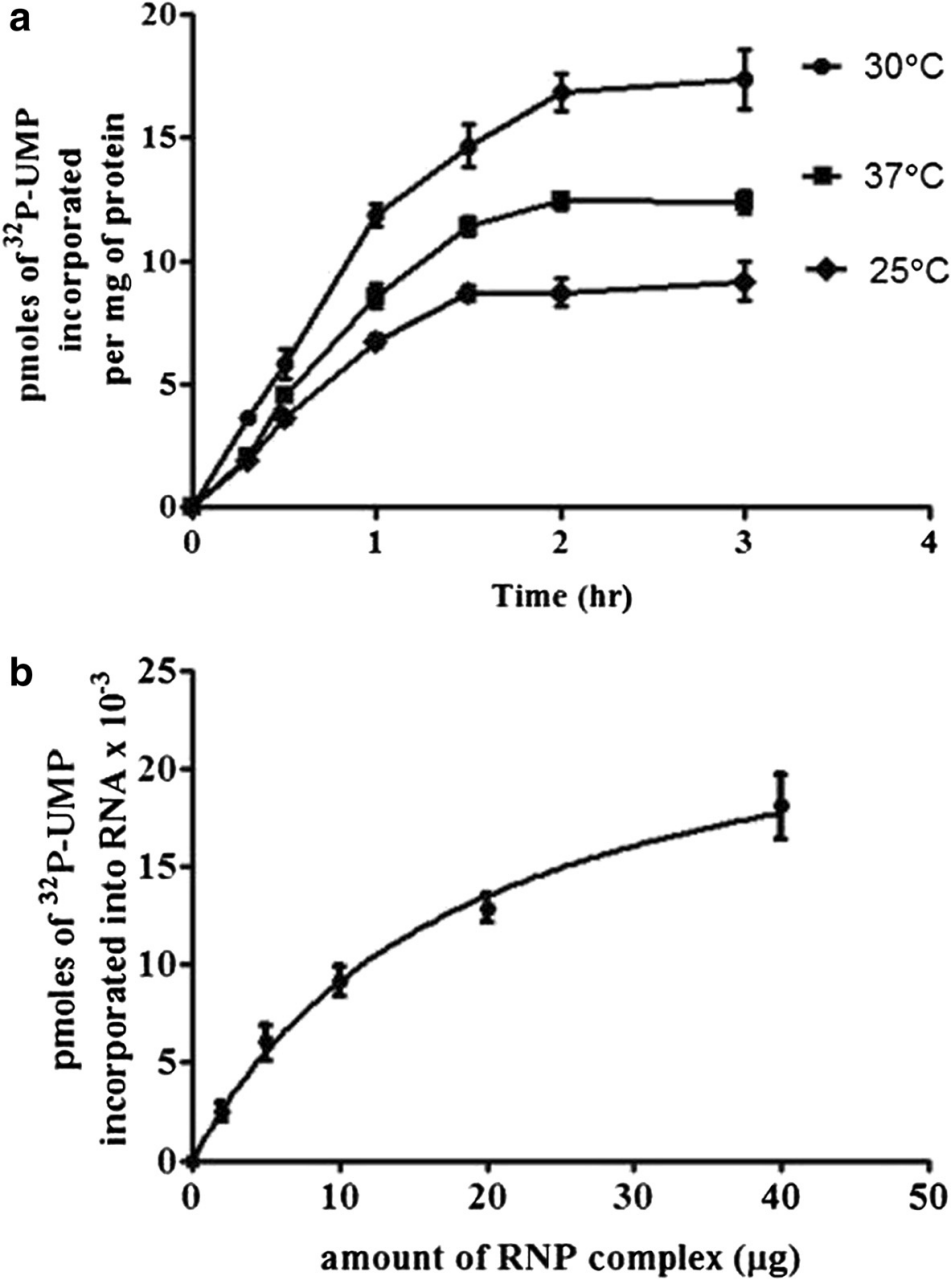
**(a) *in vitro* transcription with RNP complex-Time kinetics of RNA synthesis.** Transcription reaction mixture containing 20 μg of RNP complex purified from PPR infected Vero cells were incubated at 25, 30 and 37°C for different times. The incorporation of ^32^P-UMP into RNA was monitored by measuring TCA insoluble radioactivity. Transcription reaction in the absence of RNP complex was used as control. (**b**) *in vitro* transcription with RNP complex-dependency on protein concentration. Transcription reaction mixtures containing different amounts of RNP complex were incubated at 30°C for two hours.

The purified RNP complex was tested for its ability to synthesize viral mRNA *in vitro*. Incubation of purified RNP complex with α-^32^P-UTP and other NTPs resulted in a time dependent increase in incorporation of α-^32^P-UMP into TCA insoluble radioactivity (Figure 2a) suggesting that the purified RNP complex is active and is able to synthesize mRNA. RNP mediated transcription was maximal at 2 h of incubation at 30°C. The *in vitro* RNA synthesis depended on viral proteins since RNA synthesis increased linearly with the increasing concentration of RNP complex (Figure 2b).

### RNP complex synthesizes all the gene specific transcripts *in vitro*

**Figure 3:**
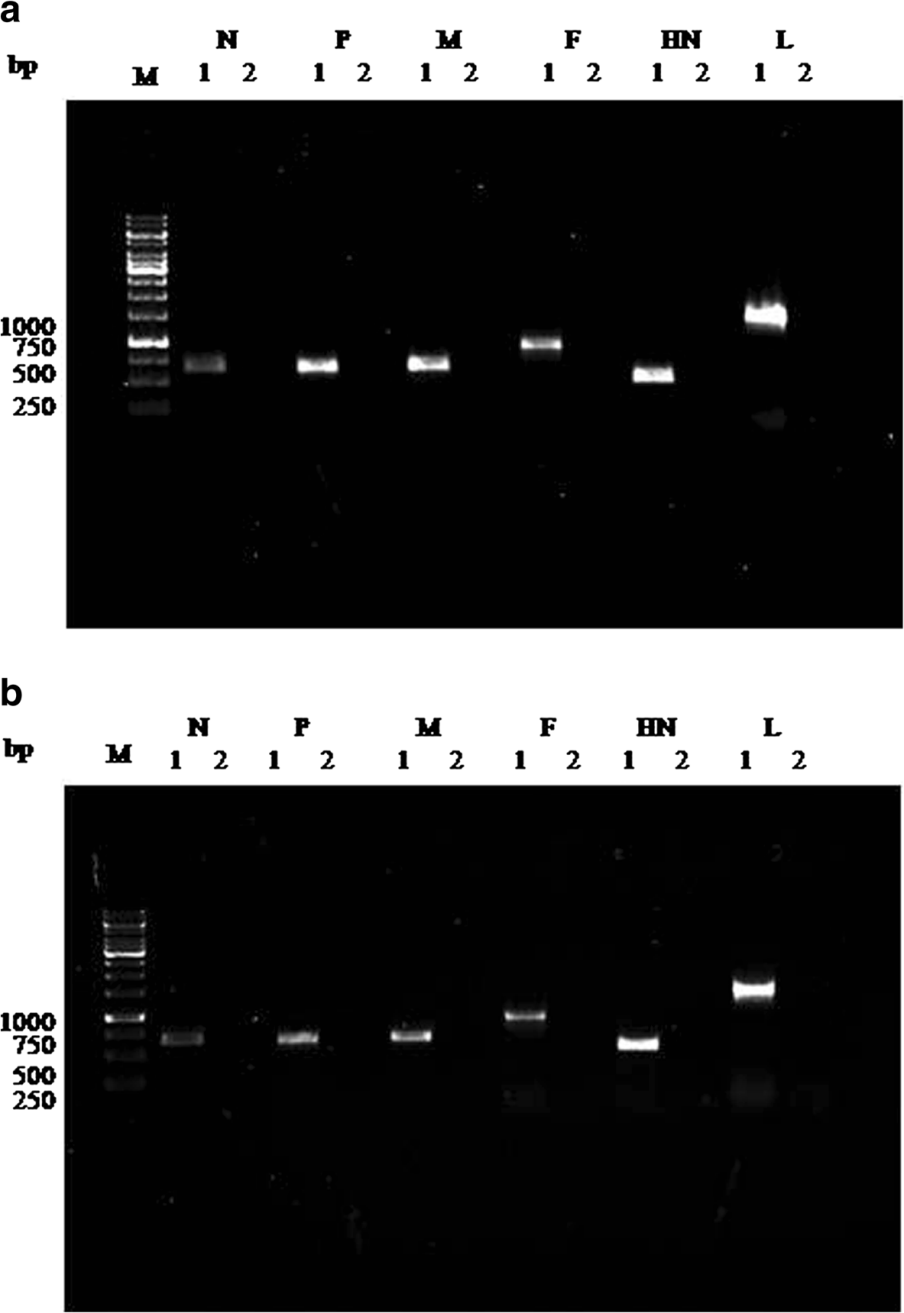
**PCR analysis of PPRV transcripts. (a)** RT-PCR analysis of in vitro transcription products from RNP of purified PPRV. Lane 1 and 2 for each gene represents the cDNA of *in vitro* transcription reaction and negative control. (**b**) RT-PCR analysis of RNA from virus infected cells. Numbers next to each band represents the size of amplicon as base pairs.

In order to investigate if RNP complex could produce authentic copies of all viral mRNA, the *in vitro* transcription reaction was carried out with unlabelled rNTPs. Isolated RNA was converted to cDNA using oligo (dT) primer. The synthesized cDNA was used for PCR amplification of all the six genes of PPRV with gene specific primers (Additional file 1: Table S1). Amplified DNA for N, P and M genes could be detected upon one round of PCR where as for F, HN and L genes, re amplification of primary PCR product was necessary in order to detect by ethidium bromide staining (Figure 3a).

The data showed that *in vitro* transcription reaction with RNP complex resulted in the synthesis of all the transcripts of PPRV. For measurement of transcripts made in virus infected cells, RNA was isolated from PPRV infected Vero cells and processed for cDNA synthesis and analyzed by PCR (Figure 3b). The size of PCR products obtained from *ex vivo* and *in vitro* experiments were identical validating the authenticity of the *in vitro* system.

### *In vitro* reconstitution of transcription with recombinant (L-P) complex and N-RNA

**Figure 4:**
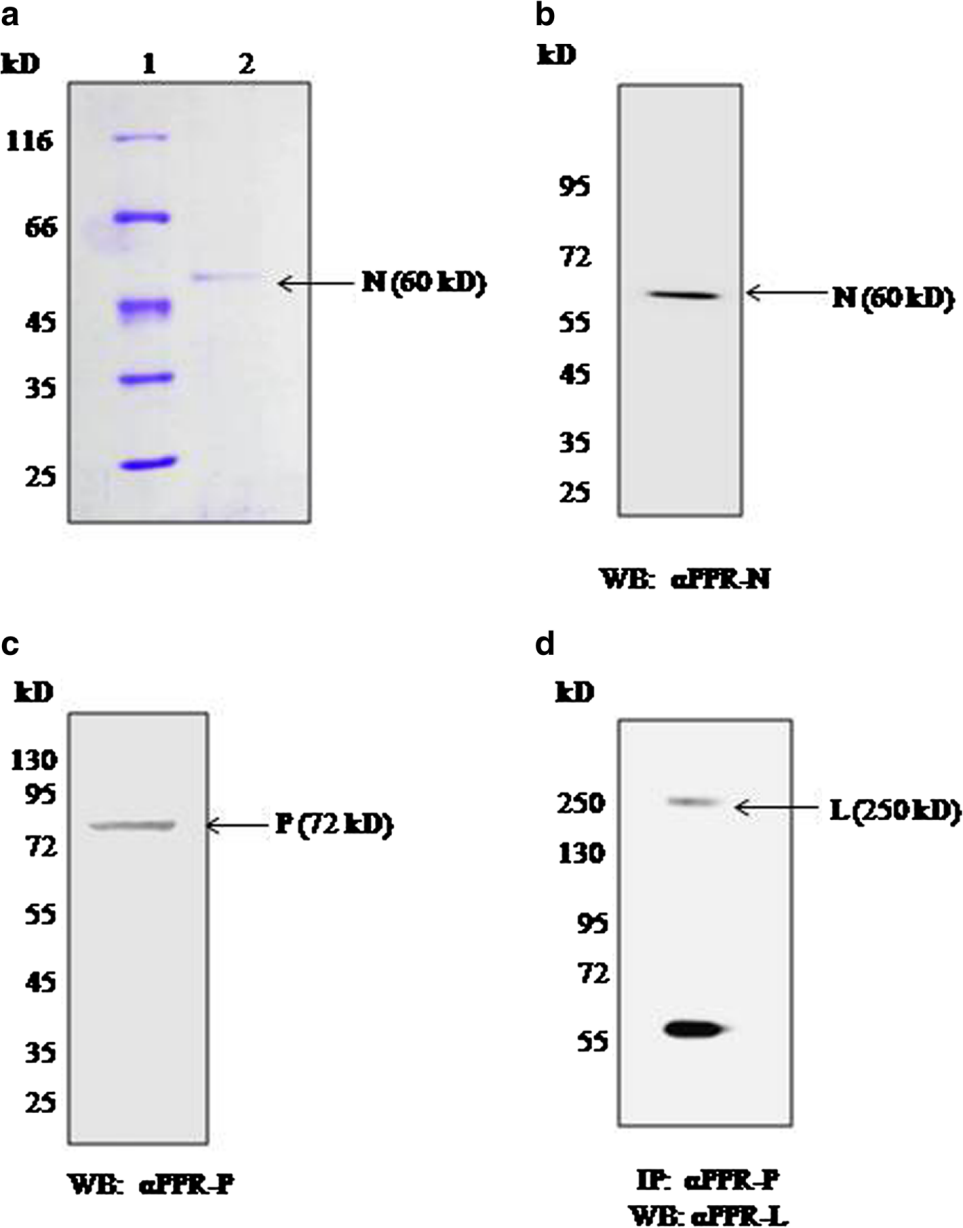
**(a) N RNA complex of PPRV was purified from virus infected vero cells.** Coomassie stained gel of purified PPR N RNA complex, lane 1 marker, lane 2 N-RNA. (**b**) Presence of N protein was confirmed by western blot using antibody against recombinant N protein of PPRV (1:4000) [19]. (**c**) 20 μg of partially purified recombinant (L-P) complex was subjected to western blot analysis for P protein with polyclonal antibody to recombinant P protein (1:4000) [7]. (**d**) To confirm L protein presence in partially purified L-P complex, 300 μg of sample was immunoprecipitated using anti P antibody and western blotting was done using an antibody against domain III of bacterially expressed L protein of PPRV (1:5000). The bottom band in the panel d represents the heavy chain of immunoglobulin used in pull down experiment.

**Figure 5:**
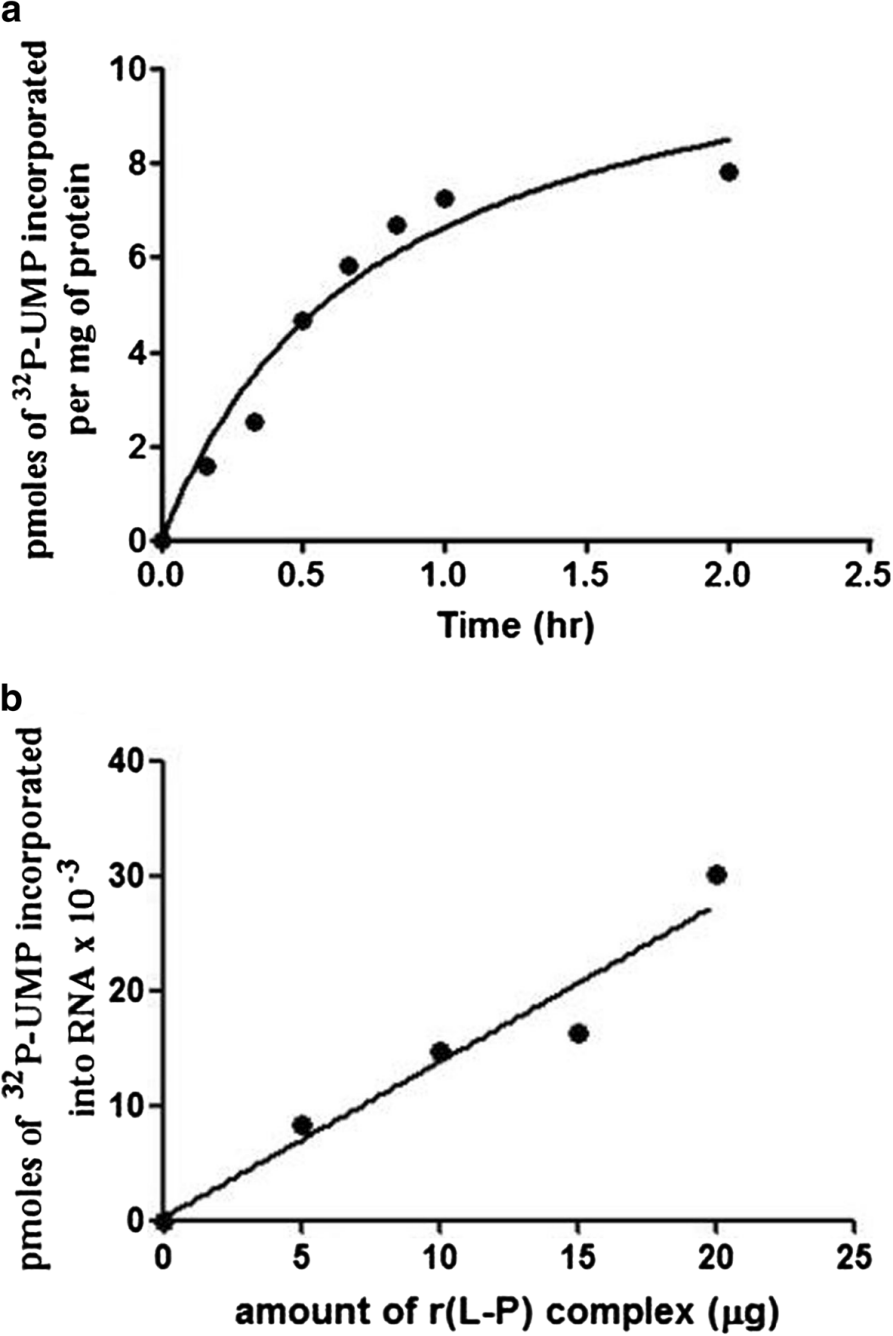
***in vitro* transcription with recombinant L-P complex.** (**a**) Time kinetics. Transcription reaction mixture containing 10μg of r(L-P) complex purified from Sf 21 cells were incubated at 30°C for the indicated time. The incorporation of ^32^P-UMP in RNA was monitored by TCA precipitation. A parallel reaction with N-RNA alone was carried out as control. Data represents the average of two independent experiments. (**b**) Enzyme concentration dependency of RNA synthesis.

To establish the *in vitro* reconstitution system for PPRV, polymerase free N-RNA complex was purified from virus infected Vero cells by CsCl density gradient centrifugation. The purified N-RNA complex contained N protein (Figure 4a). The identity of protein was confirmed by western blotting with an antibody made against recombinant N protein of PPRV (Figure 4b). Recombinant polymerase complex (L-P) was partially purified from Sf 21 cells co-infected with recombinant baculoviruses expressing L and P proteins by sucrose density gradient centrifugation. The presence of P protein in different fractions of the gradient was confirmed by western blotting with P specific antibody [7]. Fractions showing P protein were pooled. Immunoprecipitation of the r(L-P) complex with anti-P antibody followed by western blot analysis for L protein using anti-L domain III (1717–2183 aa) antibody showed the presence of L as (L-P) complex (Figure 4d). N-RNA complex was incubated with partially purified r(L-P) complex in the presence of α-^32^P-UTP and other NTPs at 30°C. The time course of incorporation of ^32^P-UMP into RNA is shown in Figure 5a. Increasing the concentration of r(L-P) complex resulted in a linear increase in the amount of ^32^P-UMP incorporation (Figure 5b). The data showed that the incubation of polymerase free N-RNA complex with r(L-P) complex results in the synthesis of mRNA in the *in vitro* transcription reaction. Reaction with N-RNA alone was unable to support RNA synthesis. In order to investigate the synthesis of all viral mRNA in the reconstituted system, isolated RNA from *in vitro* transcription reaction was converted to cDNA using oligo (dT) primer so that only authentic mRNA copies having poly (A) tail should get amplified. The cDNA was used for amplification by using gene specific PCR. The results showed (data not shown) that *in vitro* transcription reaction with purified components resulted in the synthesis of all the mRNAs of the virus.

### Quantification of transcripts synthesized by RNP and by the recombinant L-P complex

In order to assess if synthesis of mRNA follows a gradient of polarity in the reconstituted system also, *in vitro* transcription followed by quantitative RT-PCR was carried out. To be able to compare the levels of expression of different genes, primers were made to produce amplicon size very close to each other (150–160 bp). To test the efficiency of primer pairs for all the six genes of PPRV, the genomic RNA from purified virus was isolated and converted to cDNA with gene specific primer mix or random hexamer. Addition of equimolar amounts of cDNAs to real time PCR reaction mix resulted in a amplification curve whose threshold cycle values were very close to each other for the six genes.(data not shown) This result was identical for cDNA generated by random hexamer or gene specific primers. A standard curve with 10 fold serial dilution (10^1^ to 10^5^) of N gene plasmid DNA was made. The standard curve resulted in a slope of −3.42 with r^2^ value of 0.98.

**Figure 6:**
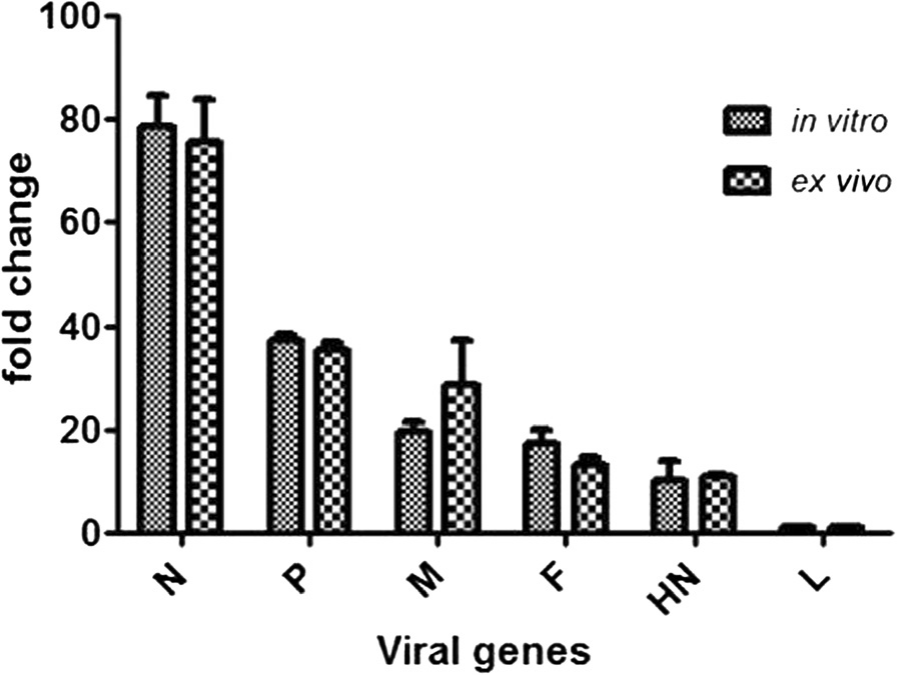
**Real time PCR analysis of viral mRNA from infected cells and *in vitro* transcription.**

**Figure 7:**
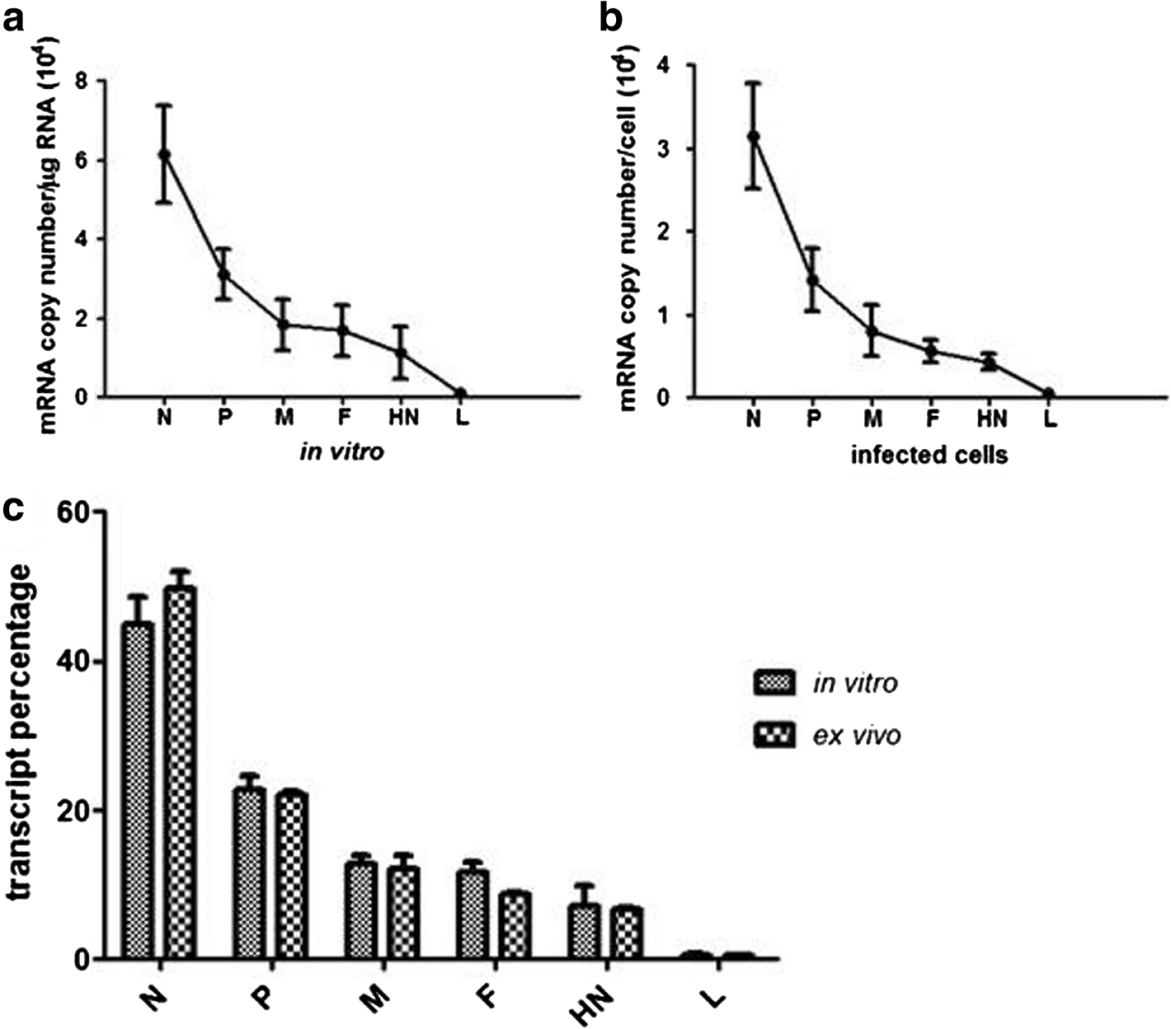
**Quantification of copy number of PPRV mRNA.** Copy numbers of individual genes of PPR virus in the (**a**) *in vitro* transcription reconstitution system and (**b**) in infected cells. (**c**) Percentage of individual transcripts of PPRV genes from total transcript.

In order to determine the relative levels of different mRNAs made in the *in vitro* reconstituted system, the cDNA made from *in vitro* transcription reaction was analyzed by real time PCR. Since the source of cDNA for all genes was one (either from *in vitro* transcription reaction or from infected cells), a master mix with all reagents except primers was made to ensure the presence of equal amount of cDNA for individual reaction. The result of real time PCR was analyzed for relative expression of different genes. The data in Figure 6 shows a gradient of polarity in transcription from 3’ end of the genome towards 5’ end with the highest transcription of N gene followed by P, M, F, HN and L gene. The transcription level for L was around 80 fold lesser than that of the N gene (N>>>P>>M>F>HN>>>L) (Figure 6). The results were normalized against the least expressed gene of the virus, L gene, and plotted as fold change. To compare the transcripts made in the *in vitro* reconstituted system with the transcript profile within the infected cells, total RNA was isolated from PPRV infected Vero cells and converted to cDNA using oligo (dT) primer. The real time PCR result is comparable to that for *in vitro* synthesized mRNA (Figure 6) suggesting the polar gradient of transcription in the infected cells as well as in the *in vitro* reconstituted system. The copy numbers of each gene synthesized in infected cells as well as in the *in vitro* reconstitution were determined. Although the quantitative values of copy number per cell were different from copy numbers made in the *in vitro* transcription reaction, the pattern of gene expression in infected cells and in the *in vitro* transcription reaction was similar (Figure 7a and b). The data given in Figure 7c shows that the N transcript represents almost 50% of the transcripts synthesized in infected cells as well as in the *in vitro* transcription reconstitution reaction.

## Discussion

The work described in the present study is the first demonstration of *in vitro* transcription reconstitution system for PPRV using RNP complex purified from infected cells as well as recombinant L-P complex purified from insect cells. The RNP complex isolated from infected cells is composed of N protein encapsidating the viral genome, L and P proteins which form the polymerase complex as shown for rinderpest virus [3] measles virus [8] and other members of mononegavirales order such as rabies virus, VSV, Sendai virus [9-11]. The RNP complex of PPRV is active in synthesizing RNA in the *in vitro* reconstituted system. Earlier work with L protein of different negative strand RNA viruses has revealed that other than polymerase function, L protein is involved in elongation, capping, cap methylation, polyadenylation and also possesses kinase activity [1,2]. With the establishment of the reconstituted system for PPRV, it would be possible to demonstrate the multifunctional activities of L protein of PPRV. In the earlier reported work with rinderpest virus, human respiratory syncytial virus and measles virus, L mRNA was not detected by Northern blotting [3,8,12]. In the present work, the synthesis of L transcript could be detected with the use of highly sensitive qPCR method. Further, to refine the *in vitro* reconstitution system for PPRV, recombinant L-P complex was expressed and purified from insect cells as has been done for Sendai and rinderpest virus [5,13]. The purified polymerase complex from insect cells when provided with N-RNA resulted in the synthesis of viral mRNA in the *in vitro* reconstituted system.

Relative quantification of virus specific mRNAs synthesized *in vitro* as well as *ex vivo* by real time PCR showed a distinct polar gradient of transcription from 3’ end to 5’ end of the genomic RNA. The results showed that N gene present at the 3’ end of the genome is the highest transcribed gene whereas L gene present at the end of genome is the least (Figure 6 and 7). These results are in accordance with results reported for HPIV-3 and measles virus using *in vitro* transcription system [14,15] as well as gene expression levels of measles virus in infected cells [16,17]. Analysis of real time PCR data revealed that the polarity of gene expression in infected cells and in the *in vitro* system follow similar pattern of gene expression. Copy number analysis of the transcripts from *in vitro* reconstitution and from infected cells although differ in their values the relative level of each gene with respect to N gene is similar in both the systems. These results support the single entry stop start model of viral transcription. The synthesis of P mRNA level is 50% of the N mRNA suggesting that after the completion of N gene transcript, the polymerase is able to successfully initiate P mRNA synthesis only once out of two attempts. Likewise polymerase is able to reach the end of the genome to synthesize L gene mRNA only once out of 75 attempts. These results are similar to the results obtained for measles virus [15,17]. The measurement of relative abundance of transcripts has revealed that 50% of the viral transcript is constituted by N gene mRNA and rest 50% by all the 5 remaining mRNAs.

The present work constitutes the first report of isolation of active transcription complex from PPRV infected cells as well as the *in vitro* reconstitution of transcription employing recombinant L and P proteins on isolated N-RNA template. Using the defined system, it is now feasible to test the post transcriptional modification activities associated with the L protein of PPRV.

## Materials and methods

### Cells and viruses

Vero cells are grown in Dulbecco’s modified essential medium (DMEM) supplemented with 10% fetal calf serum. PPRV (PPRV/Sungri/1996, obtained from IVRI, Mukteswar) isolate was used to infect Vero cells and the virus was purified according to Gopinath and Shaila [5] from culture supernatant. *Spodoptera frugiperda* (Sf 21), an insect cell line originally obtained from ATCC (USA) was supplied by National Center for Cell Sciences (NCCS), Pune, India and is being maintained in the laboratory. Recombinant baculovirus expressing P protein was earlier generated in the lab [7]. Recombinant baculovirus expressing L protein was generated from L gene clone of PPRV Tu00 (generously provided by late Dr. T. Barrett, Institute of Animal Health, Pirbright, UK) [18].

### Purification of RNP complex from infected cells

RNP complex was isolated from virus infected cells according to Ghosh et al. [3]. Briefly Vero cells monolayer were infected with PPRV at a moi of 5 and incubated for 72–84 hrs at 37°C when approximately 60-70% cytopathic effect was observed. The cells were washed with ice cold PBS and scraped off with the help of cell scraper. The cell pellet was collected by centrifugation at 2000 rpm for 10 minute at 4°C. Cells were re suspended in hypotonic lysis buffer (50mM HEPES pH 8.0, 50mM NH4Cl, 7mM KCI, 4.5 mM magnesium acetate, 1mM dithiothreitol and 0.1% Triton X-100). The cells were allowed to swell for 5 min on ice and lysed by sonication. The homogenate was clarified by centrifugation at 12,000g for 10 minute at 4°C. The supernatant fluid was collected and centrifuged through 4 ml of 50% glycerol in the lysis buffer (described above) for 2 h at 32000 rpm in a Beckman SW41 rotor at 4°C. The pellet containing RNP complexes was suspended in 1x transcription buffer (50mM HEPES [pH 8.0], 4.5mM MgOAc, 50mM NH4Cl, 7mM KCl, 1mM dithiothreitol and 1mM spermidine).

### Preparation of N-RNA template

N-RNA was prepared from PPRV infected Vero cells according to Gopinath and Shaila [5]. In brief, PPRV infected Vero cells were lysed in buffer containing 50mM Tris–HCl (pH 8.0), 150mM NaCl, 0.6% NP40, 0.4% TritonX100 and 1mM DTT. The cell lysate was clarified and EDTA was added to a final concentration of 6mM. The lysate was layered on a 20 to 40% (wt/wt) CsCl gradient and centrifuged at 200,000 X g for 2hr at 12°C in an SW41 rotor. N-RNA obtained was again purified on CsCl gradient. The N-RNA band was diluted 10 folds in TE buffer and centrifuged at 200,000 X g at 4°C for 2hr in an SW41 rotor. Finally, the N-RNA was sedimented through 40% glycerol in 50mM HEPES-KOH (pH 8.0), 50mM NaCl, 0.2% NP40 and 1mM DTT onto a 100μl cushion of 100% glycerol and stored in aliquots at −70°C at a concentration of 0.6μg/μl. The presence of N-RNA was confirmed by western blotting using antibody against recombinant PPRV N protein [19].

### Partial purification of L-P complex

Recombinant (L-P) complex of PPRV was partially purified from infected Sf 21 cells as described for rinderpest virus [5] and sendai virus [13]. Briefly Sf 21 cells (2 X 10^7^) were co-infected with recombinant baculoviruses expressing PPRV L and P proteins at a moi of 10 and 2 respectively. After 48hrs post infection cells were washed once in PBS. Cells were suspended in 4ml of lysis buffer containing 50mM Tris–HCl (pH 8.0), 500mM NaCl, 0.5% Triton X 100, 1mM DTT, 5mM MgCl2 and 1X protease inhibitor cocktail. After lysis by three rounds of freeze thaw cycles, the clarified lysate (4ml) was layered onto a linear 5 to 20% glycerol gradient (1ml of 20% glycerol and 2ml each of 5, 10 and 15% glycerol) prepared in buffer containing 50mM HEPES-KOH (pH 8.0), 150mM NH_4_Cl, 5mM MgCl2, and 1mM DTT. Gradients were centrifuged at 150,000g in SW41 rotor for 36hr at 4°C. Fractions (1.2ml) were collected from the top of the tube. 20μl fractions were electrophoresed on 10% SDS polyacrylamide gel followed by coomassie staining as well as western blotting for the detection of P protein with anti-PPRV P antibody [7].

### Reconstitution of *in vitro* transcription

*In vitro* transcription assay using either viral RNP or recombinant L-P complex was carried out according to the method described for rinderpest virus [3,5]. Briefly, *in vitro* transcription reaction was carried out in 100 μl reaction mixture having 50mM HEPES (N-2-hydroxyethylpiperazine-N'-2-ethanesulfonic acid) pH 8.0, 50mM NH_4_Cl, 7mM KCl, 4.5mM magnesium acetate, 1mM dithiothreitol, 1mM each ATP, GTP, CTP and 100 μM UTP, 10 μCi of α ^32^P-UTP (specific activity 3000 Ci/mmol, BRIT, India), 5 μg per ml of actinomycin D, and 1mM spermidine at 30°C with 20μg of RNP complex from infected cells or 10μg r(L-P) complex from insect cells. 10μl of reaction mixture was precipitated with 10% trichloroacetic acid on glass fibre filters. The filters were washed with 10% ice cold TCA three times followed by 5% ice cold TCA and finally with 70% ethanol and dried. The TCA insoluble radioactivity was measured in a liquid scintillation spectrometer.

### Analysis of transcription products by RT-PCR and real time PCR

For analysis of transcripts, *in vitro* transcription reaction was carried for two hours without radio labeled NTPs. RNA was extracted using Trizol (Sigma)-chloroform and precipitated with ethanol. The *in vitro* synthesized RNA was converted to cDNA using oligo (dT) primer and RevertAid^TM^ Premium Reverse transcriptase. For analysis of transcripts synthesized *ex vivo*, Vero cells were infected with 5 moi of PPRV and RNA was isolated by SV total RNA isolation kit, Promega as per manufacturers instruction and converted to cDNA as mentioned above. The cDNAs of *in vitro* reconstitution system were employed for real time PCR carried out using Finnzyme DyNAmoTM HS SYBR green qPCR kit. qPCR was done using Applied Biosystems 7900 HT real time PCR machine. To quantify the copy number of transcripts made *in vitro* and in infected cells, a standard curve was made by using 10 fold dilution of plasmid encoding N gene. Since all the primers were working with equal efficiency, the above standard curve was used to quantify all the six genes. The *in vitro* transcription data was expressed as copy numbers per micro gram and *ex vivo* data as copy number per cell.

## Competing interests

The authors declare that they have no competing interests.

## Authors’ contributions

MY carried out the experiments; MSS conceived the work and participated in design and coordination of the work. Both authors were involved in drafting the manuscript and approved the final manuscript.

## Supplementary Material

Additional file 1 **Table S1.** List of the primer for RT-PCR analysis. **Table S2.** List of the primers for real time PCR analysis. Click here for file
