# Supplementary material for: Establishment of an in vitro transcription system for Peste des petits ruminant virus
Source: Virol J. 2012 Dec 5;9:302. doi: 10.1186/1743-422X-9-302 (PMC3544616; doi:10.1186/1743-422X-9-302)
Supplement: Additional file 1 — Table S1. List of the primer for RT-PCR analysis. Table S2. List of the primers for real time PCR analysis. [file 1743-422X-9-302-S1.docx]

Table 1: List of the primer for RT-PCR analysis

| **Gene** | **primer** | **Sequence (5’ to 3’)** | **Location on gene** | **Amplicon size** |
| --- | --- | --- | --- | --- |
| N | N-F | CATCACGTGGTGCTGATTTAG | 350-370 | 659 |
|  | N-R | TCGCATAGCTCCAGAGGAG | 1009-991 |  |
| P | P-F | ACCCAAGCTGTGCCAGAGT | 754-772 | 651 |
|  | P-R | TGATTATGGAGCGGATGACA | 1405-1386 |  |
| M | M-F | CAGCATGGGATGTCAAAGG | 29-47 | 659 |
|  | M-R | TCTTCTAAAGTTGCCCACGTGTA | 688-656 |  |
| F | F-F | GCCAGCTACAAGGTGATGAC | 109-128 | 925 |
|  | F-R | TACAACGCATTCTGGCTGCA | 1034-1015 |  |
| HN | HN-F | AGCACAACGTGTCCTCAGTG | 638-657 | 532 |
|  | HN-R | AGGTCGAGTCTTGCATGCTT | 1170-1151 |  |
| L | L-F | tcggttgttgggttgactgat | 5131-5151 | 1421 |
|  | L-R | CTAgcctctgatgagtgcactg | 6552-6531 |  |

Table 2: List of the primers for real time PCR analysis

| **Gene** | **primer** | **Sequence (5’ to 3’)** | **Location on gene** |
| --- | --- | --- | --- |
| N | N-F | GCACAAGTCTGGATCCTTCTGG | 514-535 |
|  | N-R | TGCAATTCTGTTGCGGACTG | 672-653 |
| P | P-F | GTGGTTCAAGCAGATCCTCCAG | 388-409 |
|  | P-R | TGGACCTAGCGACATCATTGC | 538-518 |
| M | M-F | TGATTGAGGATAACGACCCCC | 173-193 |
|  | M-R | TCACTTAATCCTGCAGTGCGC | 326-306 |
| F | F-F | ACCTGATTGCAAATTGTGCGTC | 1142-1163 |
|  | F-R | TCGACTGCCGACCTGTATTGTC | 1293-1272 |
| HN | HN-F | TAGTGCCGTCTACCGATGTTCG | 1097-1118 |
|  | HN-R | TCAAGACTGACCCTGATCACCC | 1253-1232 |
| L | L-F | GACCTCATGGAACACTGATCCC | 4962-4983 |
|  | L-R | TGCGAGGCTGTTTAAAGTCGAC | 5113-5092 |
